# Use of Strawberry Tree (*Arbutus unedo*) as a Source of Functional Fractions with Biological Activities

**DOI:** 10.3390/foods11233838

**Published:** 2022-11-28

**Authors:** Diego Morales

**Affiliations:** Nutrigenomics Research Group, Department of Biochemistry and Biotechnology, Universitat Rovira i Virgili, 43007 Tarragona, Spain; diego.morales@urv.cat

**Keywords:** *Arbutus unedo*, strawberry tree, antioxidant, phenolic compounds, arbutin, antitumoral, hypoglycemic, antimicrobial

## Abstract

*Arbutus unedo*, commonly named ‘strawberry tree’ (ST), is a Mediterranean native plant that represents a relevant source of biologically active fractions and compounds. ST fruits, traditionally used with culinary and medicinal purposes, along with other components (leaves, roots, honeys, etc.), have been subjected to varied extraction procedures to obtain enriched and bioactive products. This work reviewed the scientific literature, searching for studies that evaluated the potential health implications of ST fractions and attending to the tested biological activities (antioxidant, antiproliferative, hypoglycemic, immune-modulatory, antihypertensive, antimicrobial, etc.), the part of the tree, the experimental model, the specific bioactive compounds and the selected extraction protocol. Furthermore, the strengths and weaknesses of the current state of the published evidence were critically analysed. Although in vitro results demonstrated the potential of ST fractions, further research is encouraged in order to obtain in vivo evidence (animal and clinical studies), assess additional activities (hypocholesterolemic, microbiome-modulatory), maximize the use of advanced extraction technologies, purify and isolate specific bioactive compounds and broaden the analysis investigating phenolic and non-phenolic molecules and their bioavailability.

## 1. Introduction

*Arbutus* genus belongs to the Ericaceae family and comprises 12 accepted species with different geographical distributions and interest (Table 1). Among them, *Arbutus unedo*, commonly named ‘strawberry tree’ (ST), is the most popular and investigated species due to its gastronomical and medicinal uses. ST (also called arbutus, strawberry madrone, Irish/Killarney ST) is an evergreen and flowering small tree or shrub whose fruits have been traditionally utilized in fermented and distilled beverages, jellies, jams, marmalades, sauces and vinegars and are even consumed fresh. This Mediterranean native plant can be found in Southern, Western and Central Europe, Canary Islands, Ireland, North-Eastern Africa and Western Asia, growing in regions with unusual intense dryness or frost [1,2].

ST fruits are the most exploited for culinary purposes, and they show a high carbohydrate content (70–80% dry weight) with a relevant fiber fraction (10–30% dw), constituting an interesting source of vegetal protein (1–9% dw) and lipids (2–3% dw), these percentages being dependent on the ripeness stage, location and environmental conditions (temperature, humidity, soil, etc.) [12,13]. However, other ST parts such as leaves, roots, wood and honeys have also been valued not only because of their inclusion in food and beverage preparations but also because of their content in bioactive compounds (polyphenols, lipids, proteins, secondary metabolites, etc.) that are able to exert relevant biological activities that can contribute to reducing the incidence of varied disorders (Table 2) [1,13].

Targeting the aforementioned molecules, most of the efforts carried out by food scientists and technologists have been focused on applying extraction methods to obtain functional fractions using ST raw materials (Table 3) [28].

The published works (Table 3) show that the most frequent techniques involved traditional solid–liquid extraction using different solvents—mainly, water, ethanol and methanol, but also ethyl acetate, acetone or hexane, in some cases [26,50]. In addition, advanced technologies more closely related to Green Chemistry principles have also been tested, e.g., supercritical fluid procedures or ultrasound- and microwave-assisted extractions. However, these sustainable alternatives represented a very minor percentage, and other novel approaches such as pulsed-electric fields, ionic liquids or deep eutectic solvents have not been considered yet for ST [28,35,64].

Among the investigated compounds and bioactivities, phenolics have attracted the greatest attention, particularly associated with antioxidant properties (with health and technological implications) but also with antiproliferative, hypoglycemic, anti-inflammatory, antihypertensive and antimicrobial actions [17,22,26,30,39,71]. While >90% of the works highlighted polyphenols as target compounds, a significantly lower number of publications were focused on the presence of other biologically active molecules such as ascorbic acid, tocopherols, carotenoids, fatty acids, lectins and other proteins, carbohydrates, etc. These non-phenolic compounds were particularly effective as antioxidant and antiproliferative or cytoprotective agents, and the reported activities encourage further investigation to broaden the spectrum of possibilities and efficiently exploit the versatility of ST composition [28,38,64,65].

Nevertheless, despite the promising in vitro results, just a few in vivo experiments have been carried out so far to validate the real impact of ST fractions on health. Among them, the hypoglycemic effect of roots extracts was confirmed in different rodent models [25,72,73], as well as the safety of leaf extracts administration in rats [84] and the anti-inflammatory and immune-modulatory effects in CD mice [75], but most of the activities have not been assayed in animals, and no clinical trials have been registered to date testing ST parts, extracts or isolated molecules. This lack of animal and human studies must be considered as a key point for the future to be able to formulate novel healthy products based on ST components.

In this article, the scientific literature was reviewed with the aim of identifying the recent insights into the obtention of functional fractions from ST, investigating the relevance of the different ST parts as raw materials, the described biological activities, the utilized experimental models and extraction methods (conventional and advanced) and the identification of the active molecules as well as the potential effects on human health in spite of the absence of clinical interventions.

## 2. Antioxidant Activity

When the production and accumulation of oxygen reactive species cannot be alleviated by the action of biological mechanisms in cells and tissues, the resultant imbalance is called ‘oxidative stress’, and the related damage may lead to cancer, cardiovascular, neurological, respiratory and renal diseases, among others [85]. Since the use of dietary antioxidants has been demonstrated to be effective in preventing the disturbances or restoring the balance, vegetal sources have been exhaustively investigated during recent decades to obtain potent antioxidant fractions [86,87].

ST components, particularly fruits and leaves, have been subjected to numerous and varied extraction procedures to obtain phenolics-rich fractions with high antioxidant power (Table 3). In general, solid–liquid extractions were carried out using solvents such as water, ethanol or methanol, although other solvents such as ethyl acetate, acetone or dichloromethane and more advanced technologies such as pressurized liquid extraction (PLE) were utilized for harder matrices such as roots [26,63].

Most of the works utilized methodologies that determined the ability of the phenolics-rich extracts to scavenge free radicals (DPPH•, ABTS•+, etc.), as well as other in vitro assays such as the β-carotene or crocin-bleaching assays, the measurement of lipid peroxidation inhibition, etc. Furthermore, more specific experiments were carried out by Mendes et al. (2011) [49] using human erythrocytes that were incubated with aqueous extracts obtained from ST fruits and leaves. These extracts exerted high antioxidant capacities by inhibiting oxidative hemolysis and lipid peroxidation [49]. In addition, a similar leaf fraction obtained by ultrasound-assisted water extraction also inhibited lipid peroxidation in human peripheral blood lymphocytes [59].

Apart from the already mentioned sources (fruits, leaves, roots), honeys have also been studied because of their antioxidant potential. In this sense, honeys with high levels of phenolic compounds such as arbutin and homogentistic acid demonstrated the ability to scavenge free radicals and impede cholesterol and liposomes oxidative degradation [21,61,62].

Although the main purpose of this research field is clinical and targets human health, the antioxidant potential of ST has been concurrently studied from a technological perspective. Thus, anthocyanins-rich fractions extracted from fruits using the acidified ethanol or water were tested, supplementing yoghurts [14] and wafers [41], respectively. Both experiments concluded that the addition of the extract can replace synthetic antioxidants and colorants and might lead to the design of healthy food products [14,41]. Similar results were obtained in other matrices such as cheese, sausages, pork burgers or limpet pâtés, showing that ST ingredients are promising candidates to be used in functional and novel foods [15,40,42,43].

However, despite the auspicious outcomes that have been reported, all the works have employed in vitro models to date. The validation in animal models and clinical trials is mandatory to reach clinical significance and to demonstrate the real impact of the described extracts on human health.

## 3. Antiproliferative/Antitumoral Activity

Cancer is still the second-leading cause of death worldwide, and it is accompanied by high morbidity, constituting a major concern that has motivated a vast amount of research to find and validate novel alternatives based on natural and edible products that can avoid the disadvantages of anticancer drugs such as the side effects and low specificity [87,88].

Accordingly, STs have also been included in the plethora of plant species that have been studied for this purpose. In this case, research identifying ST fruits as a source of antitumoral compounds is scarce, but a methanolic extract prepared by Guimarães et al. (2014) [32] was shown to be effective in inhibiting the growth of different tumoral cell lines, highlighting its activity against NCI-H460 (non-small lung cancer) cells and correlating the stronger potential of the ST fruit extract (other species such as *Prunus spinosa*, *Rosa micrantha* and *Rosa canina* were also tested) with its particular phenolic profile with a high abundance of galloyl derivatives [32]. Furthermore, Alexandre at al. (2020) obtained a set of extracts, subjecting the fruit residues that were discarded after distillation processes (to prepare alcoholic ST beverages) to supercritical-CO_2_ technology using ethanol as a co-solvent and a range of temperatures and pressures [64]. Those obtained at 55 °C and 100 or 300 bar were the most effective as antiproliferative agents against HT-29 (human colorectal adenocarcinoma) cells, and correlations between the activity and the content in specific phenolic compounds such as pyrogallol were observed. Moreover, the growth of HT-29 cells was also inhibited by a protein-rich product with a high content of lectins that was prepared from ST leaves [65]. As a matter of fact, leaves were utilized to generate other extracts using different solvents (water, ethanol, methanol, hexane) that were able to inhibit specific animal and human cell lines such as L20B (rat embryonal rhabdomyosarcoma), Vero (monkey kidney carcinoma), KB (human epithelial carcinoma), HeLa (human cervical epithelial carcinoma), A431 (human epidermioid carcinoma), A375 (human malignant melanoma) and RD (human embryonal rhabdomyosarcoma) [17,66,67]. Besides fruits and leaves, honeys demonstrated cytotoxic effects against human colon adenocarcinoma (HCT-116) and metastatic (LoVo) cells [19] (Table 4). Regarding the involved mechanisms of action that ST compounds exerted against cancerous cells, further investigation is needed to explain the exact actions, although the published works reported that these effects are strongly correlated with the antioxidant activity of ST phenolics [32,64]. Moreover, their ability to scavenge free radicals and other pro-oxidant molecules encourage the conduction of future studies to confirm their ability to prevent carcinogenesis in vivo. Thus, animal and human trials are still required to really understand the power of these substances to be used in chemoprevention or anticancer therapies.

## 4. Hypoglycemic Activity

Diabetes is a chronic metabolic disease primarily characterized by hyperglycemia as a consequence of a lack of insulin or resistance. Although diabetic patients are normally treated with therapies that involved drugs (metformin, insulin), novel treatments based on functional dietary ingredients can be certainly advantageous, facilitating administration and avoiding side effects [89]. Fruits, leaves and mainly roots of ST have been used within this approach to obtain hypoglycemic fractions: a methanolic extract from ST fruit with a high content in phenolic compounds inhibited α-glucosidase activity in vitro [31], as well as different ethanolic and hydroalcoholic extracts from fruits and leaves that were additionally able to inhibit α-amylase and contained significant levels of flavonoids and iridoids [55]. Regarding root extracts, pressurized water extraction produced a catechin-rich fraction that was able to inhibit α-glucosidase activity [74], and, besides the in vitro insights, other procedures using hot water resulted in antidiabetic extracts that validated their properties in vivo by inhibiting glucose absorption and enhancing glucose tolerance in healthy Sprague–Dawley rats [25] and also in healthy and diabetic Wistar rats [72,73] (Table 5).

## 5. Anti-Inflammatory/Immune-Modulatory Activity

Inflammatory responses against infection, injury, irritation, etc. are non-specific immune mechanisms that can become chronic and are linked to specific conditions such as cardiovascular disease, cancer, arthritis or allergy. Within this context, a plethora of vegetal matrices have been investigated, searching for compounds that exert immune-modulatory or anti-inflammatory properties [90].

Just a few studies have been carried out using ST components to study immune-modulatory function, and most of them utilized leaves as the main source. For instance, Moualek et al. (2016) [56] reported that an aqueous extract obtained from ST leaves showed in vitro anti-inflammatory capacity by inhibiting heat albumin denaturation and showing a protective effect of human erythrocytes membranes against induced hemolysis. The authors attributed this to the ability of the ST extract to modify the calcium influx in red blood cells [56]. The extract was rich in phenolic compounds, as well as those recovered from leaves using methanol [75,76]. In this case, the methanolic fraction inhibited the activation of STAT1 elicited by IFN-γ in MDA-MB-231 (human breast cancer) cells, human fibroblasts and human alveolar epithelial (A549/8) cells. STAT1 is a complex protein that has to be finely controlled to regulate the inflammatory process, i.e., its excessive or erroneous activation might generate exacerbated inflammatory responses [75,76]. Furthermore, this bioactive fraction inhibited STAT3 activation mediated by IL-6 in THP-1 cells (human leukemia monocytes). This activity reduction is considered as a strategy to ameliorate inflammation and was correlated in this work with the down-regulated expression of iNOS and ICAM-1 (both inflammatory genes) in A549/8 and THP-1 cells [75]. The promising in vitro results were validated in vivo in male CD mice that were subjected to carrageenan-induced pleural and lung inflammation. The pleural exudates that were collected from the group that was orally treated with the ST extract showed reduced levels of pro-inflammatory cytokines (TNF-α, IL-1β, IL-6), and the lung injury in animals was also decreased [75].

Apart from leaves extracts, ST honeys and the phenolic fractions obtained from them (using Amberlite XAD-2 resin columns for purification) exerted potent in vitro inhibitory activities against hyaluronidase. Surprisingly, honeys showed a higher inhibition percentage than their extracts, suggesting that other non-phenolic compounds are involved in the anti-inflammatory mechanism of action [21].

## 6. Antihypertensive/Vasodilatory Activity

Hypertension is a long-term condition associated with persistent blood pressure levels, and it is considered a major risk factor for cardiovascular disease, which is still the leading cause of death worldwide [91]. Most antihypertensive approaches involve the study of the renin-angiotensin system and the inhibition of its enzymes, particularly angiotensin-converting enzyme (ACE). Therefore, many works have looked for ACE-inhibitory dietary ingredients [92,93], but, surprisingly, this is not the case with *Arbutus unedo*. However, a few studies were conducted that evaluated the effect of ST extracts as vasodilatory agents or as inhibitors of platelet aggregation. In this context, Legssyer et al. (2004) [77] observed an endothelium-dependent vasorelaxant effect of ST leaves extracts obtained with water or methanol and applied to Wistar rat aorta (ex vivo) [77]. These authors also fractionated the methanolic extract using semipreparative high-performance liquid chromatography (HPLC) or caffeine addition for tannins precipitation, and it was observed that polyphenolic compounds such as oligomeric condensed tannins and catechin gallate were the most active molecules. A similar effect was observed for an aqueous roots extract [80], but the vasodilation was not achieved with ST honey [22].

In addition, leaves extracts induced the inhibition of platelet aggregation in vitro. This antiaggregant activity was noticed for an aqueous fraction, but particularly for those obtained using methanol or ethyl acetate. When the methanolic fraction was subjected to tannin precipitation, it was elucidated that tannins were mainly responsible for the inhibitory action [78]. Regarding the mechanism of action, a later study demonstrated that the inhibitory effect exerted by hot water, diethyl ether and ethyl acetate extracts (from ST leaves) was likely mediated by their high antioxidant activity (attenuating ROS production) and their inhibitory capacity against protein tyrosine phosphorylation and calcium influx in the platelets [79].

## 7. Antimicrobial/Antiviral Activity

Many plant species have been tested as a source of natural antimicrobial compounds to prevent or eliminate the presence of pathogens or non-desirable microorganisms in food products or the human body, i.e., from a technological or clinical perspective [94].

ST components (fruits, leaves, roots and honeys) have been exhaustively investigated for this purpose. Regarding fruits, an ethanolic fraction with a high content of phenolic compounds showed a strong antimicrobial activity against *Staphylococcus aureus*, *Bacillus subtilis* and *Pseudomonas aeruginosa* [30]. Later, an aqueous extract of ST fruit was added to limpet pâtés, and it showed the capacity of controlling the microbial stability of the products along 90 days of preservation, conducting a reduced microbial development compared with the non-supplemented pâtés and with no presence of pathogenic species [15]. However, ST leaves were the material that led to the highest number of studies. A great diversity of extracts (using ethanol, methanol, hot water) constitute an interesting battery of antimicrobials, inhibiting the growth of *S. aureus*, *P. aeruginosa*, *Escherichia coli*, *Enterococcus faecalis*, *Bacillus cereus* and *Mycobacterium* spp. [52,53,54,80,81]. Moreover, silver nanoparticles prepared with an aqueous ST leave extract were also able to inhibit *S. aureus*, *P. aeruginosa*, *E. coli* and *Staphylococcus epidermis* [82]. Antiviral activity was also reported in the case of an ethanolic leave extract that was effective against herpes simplex virus type-1 [17].

Roots were also subjected to extraction procedures to obtain phenolic-rich products with antimicrobial activities. Dib et al. (2013) reported a poor capacity against *S. aureus* and *P. aeruginosa* for methanolic and aqueous extracts but a moderate capacity against *E. coli* in the case of the water extract [24]. However, when advanced extraction technologies such as PLE were applied to the ST roots, the antibacterial potential was increased, as was shown for the methanolic PLE product that was highly active against *S. aureus*, *E. coli* and *Salmonella* spp. [26].

Finally, a study that evaluated ST honey reported promising antibacterial activities against *S. aureus* and *S. epidermis* and antifungal activity against *Candida* spp. [20]. However, these results were not in concordance with those obtained by Osés et al. (2020) [21], which concluded that ST honeys were not effective against bacteria or fungi. This discordance might be due to the differences in the honeys composition, and when the phenolic fractions were purified from them, the antimicrobial activity was observed. The authors attributed this to the presence of non-phenolic compounds inhibiting the antimicrobial activity in honeys or just to the lower concentration of the active compounds in the original matrix [21].

Despite the significant number of publications addressing antimicrobial properties, further research is needed to elucidate the details of the molecular mechanisms. Nevertheless, it is widely described that phenolic compounds are synthesized in response to microbial infections by plants species such as ST, being effective against a broad range of microorganisms [24]. Moreover, when ST phenolics are orally consumed, gut microbiota species (e.g., *Bifidobacterium* spp.) can utilize glycoside forms exerting β-glucosidase activities and releasing aglycones that might be more active. In this sense, the antibacterial activity of arbutin and its aglycone (hydroquinone) was investigated, and after the *Bifidobacterium* β-glucosidase action, the bioavailability and bioactivity of aglicones were increased [95].

## 8. Other Biological Activities

Apart from all the bioactivities that were previously described, other particular properties must be highlighted for ST fractions.

For instance, ST leaves were subjected to hydroalcoholic extraction and water infusion, and the resulting products were effective against lithiasis and in the prevention of calcium oxalate’s crystals formation [50]. Apparently, the mechanism of action seemed to not be directly related to the antioxidant activity of the extracts and was shown to be dependent on pH variations that estimulated the formation of soluble complexes between functional groups of the active ingredients (phenolics and, mainly, flavonoid derivatives) and calcium oxalate. The in vitro insights were promising enough to encourage further investigation to elucidate the litholytic mechanism of action and the potential applications of these fractions in the prevention or treatment of urolithiasis, a condition caused by urinary biochemical disturbances and characterized by the formation of stones in the kidneys or the urinary tract [50].

Furthermore, when leaves were macerated with ethanol, methanol or hexane, the produced infusions—particularly, the hexane fraction—were strongly active against *Leishmania major*, *Leishmania tropia* and *Leishmania infantum*, these being intracellular parasites responsible for leishmaniasis, a tropical and subtropical disease transmitted to humans by the bite of *Phlebotomus* and *Lutzomyia* insects that causes high mortality and morbidity, especially in Africa [16]. The mentioned work did not report correlations between the biological action and the concentration of the studied compounds (total phenolics and flavonoids) and suggested a possible synergistic effect of the minoritary components. Further studies are required to identify the responsible molecules [16].

An additional interest of *A. unedo* is the presence of arbutin. This phenolic compound, the glycosylated form of hydroquinone, was detected in ST, and, besides antioxidant, anti-inflammatory, antiproliferative and antimicrobial properties, it has attracted the attention of the cosmetic industry due to its potential as a tyrosinase inhibitor in the melanogenesis pathway, i.e., reducing melanin pigments formation and, therefore, being a promising candidate to be used as a skin-whitening agent correcting dark spots or blemishes. However, laboratory procedures of arbutin synthesis have already been developed and are more frequently used than arbutin extraction and purification from natural sources such as ST [96].

## 9. Conclusions, Current Limitations and Future Perspectives

This work reviewed the scientific literature directly related to *A. unedo* as a source of bioactive fractions. Considering the vast knowledge about ST and the great number of works investigating its properties, it can be concluded that the obtained results are promising enough to encourage further studies that may elucidate the responsible molecules and build stronger evidence for the development of novel functional products.

Although the previously described activities were demonstrated in vitro, validation using animal models has not been carried out yet for most of the activities. In fact, only the hypoglycemic action of root extracts was confirmed in rats and mice [25,72,73], but antioxidant, antiproliferative, anti-inflammatory, antimicrobial, antiviral, antileishmanial and anti-urolithiasis activities were evaluated in vitro or ex vivo in all cases. No clinical trials have been published to date. In addition to the above, it seems that there is an imbalance in the study of the mentioned capacities, which is mostly focused on antioxidant activity to the detriment of the others (Table 3).

Moreover, some relevant properties have not been tested yet. For instance, no studies addressing the hypocholesterolemic potential of the fractions were found. The presence of phenolics, bioactive lipids and polysaccharides, among others, and the clinical impact of hypercholesterolemia in cardiovascular health suggest that further investigations must be carried out to target ST compounds with the capability of reducing cholesterol absorption and/or biosynthesis. Similarly, the ability to modulate human gut microbiota is still a pending issue that must be studied for ST fractions, since this factor may cause relevant effects on human health status [97,98].

In order to obtain ST bioactive products, several extraction procedures were described in the literature; however, advanced technologies have been used in just a few works. These methodologies are frequently recommended because of their advantages over traditional protocols: a higher specificity, lower solvent volumes, shorter extraction times, etc. These benefits allow for one to work in concordance with the principles of Green Chemistry and lead to environmentally friendly methods linked to sustainability. Therefore, although some of these techniques were applied to ST (supercritical fluids, pressurized liquids, ultrasound and microwave extractions), their utilization, as well as that of other novel ones (pulsed-electric fields, ionic liquids, deep eutectic solvents, solvent-free-microwave extraction, etc.), must be strongly advised [99].

Other weakness of the current status of the art is the lack of works carrying out the purification and isolation of single compounds (except for some phenolic compounds such as arbutin) that might determine compound–activity correlations. A large majority of the studies are only targeting phenolic compounds, without taking into consideration the great diversity of molecules that might exert relevant biological activities (proteins and peptides, carbohydrates, glycoproteins, lipids, secondary metabolites, etc.).

Moreover, since the obtained products may be dietary ingredients, bioaccesibility and bioavailability assays must be conducted, also defining the effect of the food matrix, digestion, interaction with microbiota, etc.

In summary, some points must be further investigated: animal and clinical studies to evaluate biological activities; the assessment of other properties such as hypocholesterolemic or microbiome-modulatory properties; the use of advanced extraction technologies; the purification and isolation of single compounds; the deeper investigation of non-phenolic components; and bioaccesibility and bioavailability studies. The resulting insights may be crucial in validating the auspicious results that were reviewed and described in this work.

## Figures and Tables

**Table 1:** List of accepted *Arbutus* species, geographical distribution and main parts of interest.

*Arbutus* Species	Geographical Distribution	Main Parts of Interest	References
*A. unedo*	Europe (South, West, Central), Africa (North-East), Asia (East), Canary Islands, Ireland	Fruits, leaves, roots, honeys	[1,2]
*A. andrachne*	Europe (South-East, East), Asia (West)	Fruits, leaves	[3,4]
*A. canariensis*	Canary Islands	Fruits	[5]
*A. pavarii*	Lybia	Fruits, honeys, bark	[6]
*A. arizonica*	USA, Mexico	Fruits	[2]
*A. madrensis*	Mexico	-	[2,7]
*A. menziesii*	USA, Mexico, Canada	Fruits	[8]
*A. occidentalis*	Mexico	-	[2]
*A. tessellata*	Mexico	-	[9]
*A. xalapensis*	USA, Mexico, Nicaragua, El Salvador, Guatemala, Honduras	Bark, wood	[10]
*Arbutus* × *andrachnoides* (*A. unedo* × *A. andrachne*)	Greece, Turkey, Cyprus	Fruits	[11]
*Arbutus* × *androsterilis* (*A. unedo* × *A. canariensis*)	Canary Islands	Fruits	[5]

**Table 2:** Traditional uses, biological activities and potential health implications (related disorders whose risk may be reduced) of the main parts of *Arbutus unedo*.

Part	Main/Traditional Uses	Tested Biological Activities	Potential Health Implications	References
Fruit	Food, beverages, medicinal	Antioxidant, hypoglycemic, antimicrobial, antiproliferative	Diabetes, hypertension, metabolic syndrome, cancer	[14,15]
Leaves	Infusions, medicinal	Antioxidant, anti-inflammatory, hypoglycemic, antimicrobial, antiviral, antiproliferative, antihypertensive, anti-urolithiasis, antileishmanial	Diabetes, hypertension, metbolic syndrome, viral infections (papilloma virus), urolithiasis, leishmaniasis	[16,17]
Flower	Medicinal	Antioxidant	Oxidative stress-related disorders	[18]
Honey	Food, medicinal	Antioxidant, anti-inflammatory, antimicrobial, antiproliferative, antihypertensive	Hypertension, metabolic syndrome, cancer	[19,20,21,22]
Bark/wood	Firewood, tools, ornamental	Antioxidant	Oxidative stress-related disorders	[23]
Root	Medicinal, red dye (textile industry)	Antioxidant, hypoglycemic, antimicrobial, antihypertensive	Diabetes, hypertension, metabolic syndrome	[24,25,26,27]

**Table 3:** Biological activities of *Arbutus unedo* extracts tested in different experimental models.

Activity	Raw Material	Experimental Model	Bioactive Compound(s)	Extraction Method	Reference
Antioxidant	Fruits	Radical scavenging assays	Phenolic compounds	Delipidation + Ultrasound-assisted extraction (Ethyl acetate; 80% methanol)	[29]
				80% ethanol; 80% methanol extraction	[30]
				80% methanol extraction	[31]
				80 and 100% methanol extraction	[32]
			Phenolic compounds (gallic acid, cyanidin 3-glucoside), ascorbic acid	-	[33]
			Phenolic compounds, fatty acids, α-tocopherol	96% ethanol extraction	[34]
			Galloyl hexoside, 5-O-galloylquinic acid	Supercritical-CO_2_ extraction (40, 55, 70 °C)	[35]
		Radical scavenging and β-carotene bleaching assays	Phenolic compunds, ascorbic acid	Ethanol extraction	[36]
			Phenolic compounds (gallocatechol, catechin)	Ethanol extraction (Ultra-turrax homogeneization, 25 °C)	[37]
		Radical scavenging, β-carotene bleaching and lipid peroxidation inhibition assays	Phenolic compounds, ascorbic acids, tocopherols, carotenoids	-	[38]
		Radical scavenging, β-carotene and crocin bleaching assays	Phenolic compounds, carbohydrates	Maceration (20–90 °C), microwave- (50–145 °C) and ultrosound-assisted (30–35 °C) extraction (ethanol–water mixtures)	[28]
		Radical scavenging, β-carotene bleaching and lipid peroxidation inhibition assays (bread fortification)	Catechin	23% ethanol extraction	[39]
		Radical scavenging assays (yoghurt fortification)	Anthocyanins	Acidified water extraction	[14]
		Radical scavenging assays (cheese fortification)	Phenolic compounds	Water infusion	[40]
		Radical scavenging assays (wafers fortification)	Anthocyanins	80% acidified ethanol (90 °C)	[41]
		Food oxidative stability (sausages fortification)	Phenolic compounds	Ethanol extraction	[42]
		Lipid oxidation (limpet pâté)		Water extraction	[15]
		Lipid oxidation (pork burgers)	-	Ethanol extraction	[43]
		Protein oxidation (pork burgers)		Ethanol extraction	[44]
	Fruits, leaves	Radical scavenging assays	Phenolic compounds	80% methanol extraction	[45]
				Ultrasound-assisted extraction (70% ethanol)	[46]
				Ultrasound-assisted extraction (80% methanol)	[47]
			Phenolic compounds, ascorbic acid, β-carotene	80% ethanol	[48]
		Human erythrocytes	Phenolic compounds	Water infusion (100 °C)	[49]
	Fruits, flowers	Radical scavenging and β-carotene bleaching assays		Water, ethanol and methanol extraction	[18]
	Leaves	Radical scavenging assays		Delipidation + hot 80% ethanol extraction or water infusion (100 °C)	[50]
				Ultrasound-assisted extraction (50% methanol)	[51]
				Ethanol, methanol, diethyl ether extraction	[34]
				Delipidation + methanol extraction	[52]
				Water infusion (100 °C)	[53]
			Arbutin, phenolic compounds (hydroquinone)	Ethanol, water extraction (50 °C)	[54]
			-	Ethanol maceration	[17]
		Radical scavenging and β-carotene bleaching assays	Phenolic compounds, iridoids	Maceration, Soxhlet and ultrasound-assisted extraction (ethanol)	[55]
		Radical scavenging assays and inhibition of β-carotene peroxidation	Phenolic compounds	Water maceration	[56]
		Radical scavenging assays, *Saccharomyces cerevisiae* strains		50% Ethanol extraction, in vitro digestion	[57]
		Radical scavenging, crocin-bleaching and liposome-accelerated oxidation assays (teas)	Phenolic compounds (galloylquinic acid derivatives and myricitrin)	Addition of boiling water (decoction) (100 °C)	[58]
		Human peripheral blood lymphocytes		Ultrasound-assisted extraction (water)	[59]
	Honeys	Radical scavenging assays	Phenolic compounds	-	[60]
					[22]
			Phenolic compounds (arbutin)	Amberlite resin purification and methanol extraction	[21]
				-	[61]
		Radical scavenging, cholesterol, liposome oxidation assays	Homogentistic acid		[62]
	Wood, stalks, leaves	Radical scavenging assays	Phenolic compounds	60% acetone, 95% ethanol extraction	[23]
	Roots		Phenolic compounds (catechin)	Ethyl acetate or methanol extraction (100 °C)	[63]
			Phenolic compounds (flavonoids)	High-pressure solvent extraction (water, methanol, ethyl acetate, dichloromethane)	[26]
Antiproliferative/antitumoral	Fruits	Human breast adenocarcinoma (MCF-7), non-small cell lung cancer (NCI-H460), colon carcinoma (HCT-15), cervical carcinoma (HeLa) and hepatocellular carcinoma (HepG2) cells	Phenolic compounds	80 and 100% methanol extraction	[32]
	Fruit residues (after fermentation and distillation)	Human colorectal adenocarcinoma (HT29)	Phenolic compounds, fatty acids esters, terpenes	Supercritical-CO_2_ extraction (35, 45, 55 °C), 50% ethanol extraction, Soxhlet extraction (hexane)	[64]
	Leaves		Proteins (lectins)	Protein extraction	[65]
		Human epithelial carcinoma cells (KB)	-	Hot water extraction (80 °C)	[66]
		Human cervical epithelial carcinoma (HeLa), human epidermioid carcinoma (A431), human malignant melanoma (A375) cells		Ethanol maceration	[17]
		Human embryonal rhabdomyosarcoma cancerous cells (RD), rat embryonal rhabdomyosarcoma cancerous cells (L20B), monkey kidney cancerous cells (Vero)	Phenolic compounds	Hexane, ethanol and methanol extraction	[67]
	Honeys	Human colon carcinoma (HCT-116) and LoVo cells	Phenolic compounds, proteins	-	[19]
			Phenolic compounds (phenolic acids, flavonols)		[68]
			Phenolic compounds		[19,69]
	Axillary buds, leaves fragments	Murine B6-F10 melanoma cells	Anthocyanins	Cell culture (MS medium, B5 medium)	[70]
	Whole plant	Human osteosarcoma cells (U2OS)	Phenolic compounds (arbutin)	Ultrasound-assisted extraction (50% methanol)	[71]
Hypoglycemic	Fruits	α-glucosidase inhibition test	Phenolic compounds	80% methanol extraction	[31]
	Fruits, leaves	α-glucosidase and α-amylase inhibition tests	Phenolic compounds, iridoids	Maceration, Soxhlet and ultrasound-assisted extraction (ethanol)	[55]
	Roots	C57BL/6JRj mice, Sprague–Dawley rats	-	Hot water extraction (100 °C)	[25]
		Wistar rats			[72]
					[73]
		α-glucosidase inhibition test	Catechin	Pressurized liquid extraction (water, 100 °C, 10 MPa)	[74]
Anti-inflammatory/immune-modulatory	Leaves	Human red blood cells	Phenolic compounds	Water maceration	[56]
		Human alveolar epithelial (A549/8) cells, human leukemya monocyes (THP-1) and CD mice		Methanol extraction	[75]
		Human breast cancer (MDA-MB-231) cells and human fibroblasts			[76]
	Honeys	Hyaluronidase inhibition assay	Phenolic compounds (arbutin)	Amberlite resin-purification and methanol extraction	[21]
Antihypertensive/vasodilatory	Leaves	Aorta isolation from Wistar rats	Tannins and catechin gallate	Water and methanol extraction; tannins precipitation by caffeine addition	[77]
		Platelet aggregation	Tannins	Hot water (100 °C), methanol and ethyl acetate extraction; tannins precipitation by caffeine addition	[78]
			-	Hot water (100 °C), diethly ether, ethly acetate extraction	[79]
	Roots	Aorta isolation from Wistar rats		Hot water extraction (100 °C)	[27]
Antimicrobial	Fruits	Microbiological analyses	Phenolic compounds	80% ethanol extraction	[30]
		Microbiological analyses (limpet pâté)		Water extraction	[15]
	Leaves	Microbiological analyses		Water infusion (100 °C)	[53]
				Delipidation + methanol extraction	[52]
				Ultrasound-assisted extraction (50% ethanol)	[80]
			Phenolic compounds (hydroquinone, arbutin)	Ethanol, water extraction (50 °C)	[54]
			-	Ethanol and hot water extraction (100 °C)	[81]
		Microbiological analyses (silver nanoparticles)		Hot water extraction (100 °C)	[82]
	Roots	Microbiological analyses	Phenolic compounds	Methanol and water extraction	[24]
			Phenolic compounds (flavonoids)	High-pressure solvent extraction (water, methanol, ethyl acetate, dichloromethane)	[26]
	Honeys			Amberlite resin purification and methanol extraction	[21]
				Pentane, dichloromethane, butanol extraction	[20]
Antiviral	Leaves	Anti-herpes simplex virus type-1 assay	-	Ethanol maceration	[17]
Antileishmanial	Leaves	Anti-promastigote activity assays	Phenolic compounds	Methanol, ethanol and hexane maceration	[16]
Anti-urolithiasis	Leaves	Inhibition of calcium oxalate’s crystallization	Phenolic compounds, tannins, saponins, sterols, polyterpenes, alkaloids	Water and ethanol extraction (100 °C)	[50,83]

**Table 4:** Cell models and tested concentrations of ST extracts to evaluate in vitro biological activities.

Evaluated Activity	Cell Model	Tested Concentration(s)	ST Administered Product	Reference
Antioxidant	Human erythrocytes	50, 75, 100 µg/mL	Fruit aqueous extract	[49]
		0.4, 0.8, 1.6 mg/mL	Leaves aqueous extract	
	*Saccharomyces cerevisiae*	250 µg gallic acid-equivalents/mL	Leaved hydroethanolic extract (after in vitro digestion)	[57]
	Human peripheral blood lymphocytes	11.4, 200, 400 µg/mL	Leaves aqueous extract	[59]
Antiproliferative/antitumoral	MCF-7, NCI-H460, HCT-15, HeLa, HepG2	25–400 µg/mL	Fruit methanolic extract	[32]
	HT29	0.125–4 mg/mL	Fruit (residue) supercritical, hydroethanolic and hexane extracts	[64]
	KB	0.05, 0.1, 0.2 mg/mL	Leaves aqueous extract	[66]
	HeLa, A431, A375	1.56–200 µg/mL	Leaves ethanolic extract	[17]
	RD, L20B, Vero	3.5–250 µg/mL	Leaves ethanolic, methanolic and hexane extracts	[67]
	HCT-116	3–20 mg/mL	Honeys	[19,68,69]
	LoVo	10–60 mg/mL		
	U202	50, 100 µg/mL	Hydromethanolic whole plant extracts	[71]
Anti-inflammatory/immune–modulatory	Human red blood cells	50–500 µg/mL	Leaves aqueous extract	[56]
	A549/8, THP-1	0.9–59.2 µg gallic acid-equivalents/mL	Leaves methanolic extract	[75]
	MDA-MB-231	0.02–29.6 µg gallic acid-equivalents/mL		[76]
	Human fibroblasts	3.7–14.8 µg gallic acid-equivalents/mL		

**Table 5:** Dosage and administration time of ST extracts for testing biological activities in vivo.

Evaluated Activity	Animal Model	Dosage	Administration	ST Administered Product	Reference
Hypoglycemic	Sprague–Dawley rats (males and females)	0.3 and 2 g/kg	Daily at 10:00 h a.m., 6 weeks, intragastric gavage	Root aqueous extract	[25]
	Wistar rats (males)	0.5 g/kg	30 min before glucose administration, oral administration		[72]
	Wistar rats (males and females)	0.15 g/kg			[73]
Anti-inflammatory/immune modulatory	CD mice (males)	0.02 g (gallic-acid equivalents)/kg	1 h before carragenan and saline administration, oral administration	Leaves methanolic extract	[75]

## Abbreviations

Strawberry tree (ST), Ante Meridiem (AM), pressurized-liquid extraction (PLE), high-performance liquid chromatography (HPLC), angiotensin-converting enzyme (ACE), interleukin (IL), tumor necrosis factor (TNF).
